# Ultra-bright and highly efficient inorganic based perovskite light-emitting diodes

**DOI:** 10.1038/ncomms15640

**Published:** 2017-06-07

**Authors:** Liuqi Zhang, Xiaolei Yang, Qi Jiang, Pengyang Wang, Zhigang Yin, Xingwang Zhang, Hairen Tan, Yang (Michael) Yang, Mingyang Wei, Brandon R. Sutherland, Edward H. Sargent, Jingbi You

**Affiliations:** 1 Key Laboratory of Semiconductor Materials Science, Institute of Semiconductors, Chinese Academy of Sciences, Beijing 100083, China; 2 College of Materials Science and Opto-electronic Technology, University of Chinese Academy of Sciences, Beijing 100049, China; 3 Department of Electrical and Computer Engineering, University of Toronto, 35 St George Street, Toronto, Ontario, Canada M5S 1A4; 4 College of Optical Science and Engineering, Zhejiang University, Hangzhou 310027, China

## Abstract

Inorganic perovskites such as CsPbX_3_ (X=Cl, Br, I) have attracted attention due to their excellent thermal stability and high photoluminescence quantum efficiency. However, the electroluminescence quantum efficiency of their light-emitting diodes was <1%. We posited that this low efficiency was a result of high leakage current caused by poor perovskite morphology, high non-radiative recombination at interfaces and perovskite grain boundaries, and also charge injection imbalance. Here, we incorporated a small amount of methylammonium organic cation into the CsPbBr_3_ lattice and by depositing a hydrophilic and insulating polyvinyl pyrrolidine polymer atop the ZnO electron-injection layer to overcome these issues. As a result, we obtained light-emitting diodes exhibiting a high brightness of 91,000 cd m^−2^ and a high external quantum efficiency of 10.4% using a mixed-cation perovskite Cs_0.87_MA_0.13_PbBr_3_ as the emitting layer. To the best of our knowledge, this is the brightest and most-efficient green perovskite light-emitting diodes reported to date.

## Abstract (web summary)

Hybrid organic-inorganic perovskites are garnering attention for light emitting diode (LED) applications. Employing a thin hydrophilic insulating polymer, Zhang *et al*. report LEDs exhibiting a brightness of 91,000 cd m^−2^ and external quantum efficiency of 10.4% using a mixed-cation perovskite.

Organic–inorganic perovskites have received extensive attention in recent years in view of their attractive electrical and optical properties. Solution-processed perovskite solar cells have demonstrated a high certified power conversion efficiency of 22.1%, which is comparable to photovoltaics made from traditional inorganic semiconductor materials such as Si, CIGS and CdTe (refs 1, 2, 3, 4, 5, 6, 7, 8). In addition, they have been utilized as efficient low-threshold gain media in optically pumped lasers^9^,^10^. Perovskite materials exhibit high photoluminescence quantum yield (PLQY, >90% in solution for nanocrystals) and high colour purity with narrow emission linewidths <20 nm (refs 11, 12, 13, 14, 15). These features make them promising candidates as new materials for light-emitting diodes (LEDs).

Electroluminescence (EL) from trihalide organic–inorganic perovskite-based LEDs (PeLEDs) was first reported in 2014 (ref. 16). The peak brightness of these LEDs was of order 300 cd m^−2^ at green wavelengths, and the external quantum efficiency (EQE) from CH_3_NH_3_PbI_3*−x*_Cl_*x*_ (754 nm emission, red) and CH_3_NH_3_PbBr_3_- (517 nm emission, green) based LEDs were 0.76 and 0.1%, respectively^16^. By interface engineering and perovskite layer optimization, the EQE was increased to over 3% (refs 17, 18, 19, 20, 21, 22, 23, 24, 25, 26, 27). A breakthrough in organic–inorganic PeLEDs was achieved by controlling the crystallization process of CH_3_NH_3_PbBr_3_ by adopting a nanocrystal pinning method. As a result, a dense film with small crystal domains (<100 nm) was obtained, effectively confining charge carriers. These devices demonstrated an impressive EQE of 8.53% (ref. 28). More recently, by confining electrons and holes to two-dimensional (2D) perovskites^29^, Yuan *et al*. and Wang *et al*. obtained near-infrared EQEs of 8.8 and 11.7%, respectively^30^,^31^. Combined with nanocrystal pinning and 2D perovskites, Rand *et al*. also achieved close to 10% EQE of organic–inorganic LEDs^32^.

Compared with monovalent organic cation-based lead-halide perovskites, all inorganic perovskites exhibit improved thermal stability and more efficient PL, making them attractive in a number of optoelectronic device applications^14^,^15^,^33^,^34^. Inorganic perovskites such as CsPbX_3_ (X=Cl, Br, I) have attracted great attention due to their improved thermal stability and higher PLQY in comparison with organic-cation perovskites. Recently, CsPbX_3_ nanocrystals have been successfully synthesized and used as emitting materials for LEDs^14^,^15^. CsPbX_3_ thin film-based LEDs have also been demonstrated^34^. However, the EQE of the LEDs based on these materials remain <1% (refs 15, 34). During revising of this manuscript, Li *et al*. and Ling *et al*. both reported inorganic CsPbBr_3_ LEDs with about 6% EQE by surface passivation of perovskite nanocrystals and controlling perovskite thin film morphology, respectively^35^,^36^. There is still much more room for improvement in brightness and efficiency. The low efficiency may arise from high leakage current due to poor morphology (high density of pinholes), significant non-radiative recombination at the interface of perovskite/injection layers and within the perovskite layer itself and charge injection imbalance^15^,^34^.

In this report, we achieved high-quality dense CsPbBr_3_ perovskite thin films by incorporating a small amount of organic methylammonium cation into the lattice and by using a hydrophilic insulating polymer interface layer on top of the ZnO electron-injecting electrode. We fabricated high performance mixed-cation perovskite LEDs with an active layer composition of Cs_0.87_MA_0.13_PbBr_3_. These LEDs exhibited a peak brightness of 91,000 cd m^−2^ and a peak EQE of 10.43%. This represents the brightest and most-efficient green perovskite LEDs reported to date^28^,^30^,^31^.

## Results

### Morphology of CsPbBr_3_ films

**Figure 1:**
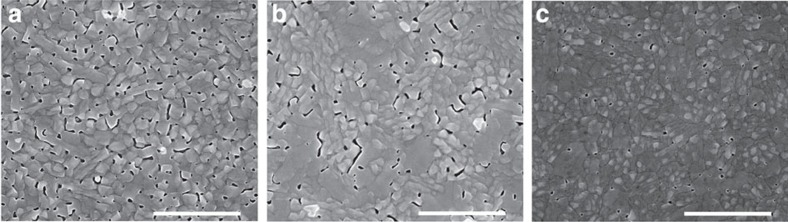
Morphology of CsPbBr_3_ films deposited under different conditions. (**a**–**c**) Planar SEM images of CsPbBr_3_ deposited on ZnO, ZnO/PVP and Cs_0.87_MA_0.13_PbBr_3_ on ZnO/PVP, respectively, here PVP is polyvinyl pyrrolidine. The scale bar is 2 μm in all images.

CsPbBr_3_ perovskite thin films were fabricated by spin-coating a CsBr:PbBr_2_ precursor from dimethyl sulfoxide (DMSO) onto substrates, followed by annealing at 100 °C for 20 min to remove residual solvent and to induce perovskite crystallization. A high ratio of CsBr:PbBr_2_ (2.2:1) precursor solution was used to guarantee the formation of pure phase CsPbBr_3_, while the excess CsBr would be readily precipitated during solution stirring^33^. As shown in Fig. 1a, the perovskite film directly deposited onto the electron-injection layer of ZnO exhibited a high density of pinholes. To improve the surface morphology, we first introduced a thin hydrophilic insulating polymer, polyvinyl pyrrolidine (PVP), between the ZnO and perovskite layers. The density of pinholes was largely reduced by inserting the PVP intermediate layer, as shown in Fig. 1b. Real-time contact angle results showed that PVP-modified ZnO films have increased hydrophilicity (Supplementary Fig. 1). As a result, the PVP-modified substrate has better wetting of the hydrophilic perovskite precursor solution, leading to uniform growth of perovskite films with reduced pinholes. Although the perovskite film surface coverage was significantly improved by introducing the PVP intermediate layer, there was still an appreciable density of pinholes. To improve further the morphology of the perovskite film, we added a small amount of CH_3_NH_3_Br (MABr) into the precursor solution. We hypothesized that molecular pinning would help reduce pinholes by better controlling the crystallization kinetics of the CsPbBr_3_ films^37^,^38^. Figure 1c shows that the morphology of the perovskite film was improved considerably once we did add MABr, leading to now a negligible density of pinholes, which could be good for reducing current leakage in LEDs.

### Crystal and band structure of CsPbBr_3_ films

We characterized the crystal structure of CsPbBr_3_ films deposited on bare ZnO and ZnO/PVP substrates, and on ZnO/PVP with the MABr additive. X-ray diffraction pattern of these three films were almost identical, with all crystallographic signatures matching that of the pure CsPbBr_3_ phase (Supplementary Fig. 2). The band structure of CsPbBr_3_ films with and without the MABr additive were determined using ultraviolet photoelectron spectroscopy (UPS; Supplementary Fig. 3, Supplementary Table 1) in combination with linear absorption measurements (Supplementary Fig. 4). The conduction and valence band relative to vacuum of CsPbBr_3_ with MABr are located at −3.37 and −5.71 eV, respectively. This could form a good alignment with electron-injecting layer such as ZnO (−3.84 eV; Supplementary Fig. 5) and hole-injecting such as CBP (−6.0 eV)^39^ (Fig. 3b).

### Chemical states of CsPbBr_3_ films

We also carried out X-ray photoelectron spectroscopy (XPS) measurements on CsPbBr_3_ films with and without the MABr additive (Supplementary Figs 6 and 7). The Pb 4f core level from pure CsPbBr_3_ can be fit to four peaks (Supplementary Fig. 7). Two main peaks are located at 138.9 eV (Pb 4*f*_7/2_) and 142.8 eV (Pb 4*f*_5/2_), which correspond to Pb–Br bonding^28^,^40^. Two additional weaker peaks at 137.1 and 141.9 eV can be attributed to Pb metallic states^28^,^40^. After incorporating MABr into the CsPbBr_3_ lattice, only Pb–Br peaks were found, indicating that Pb metallic states, which are known to function as non-radiative recombination centres^28^,^40^, have been suppressed.

### Photoluminescence of CsPbBr_3_ films

**Figure 2:**
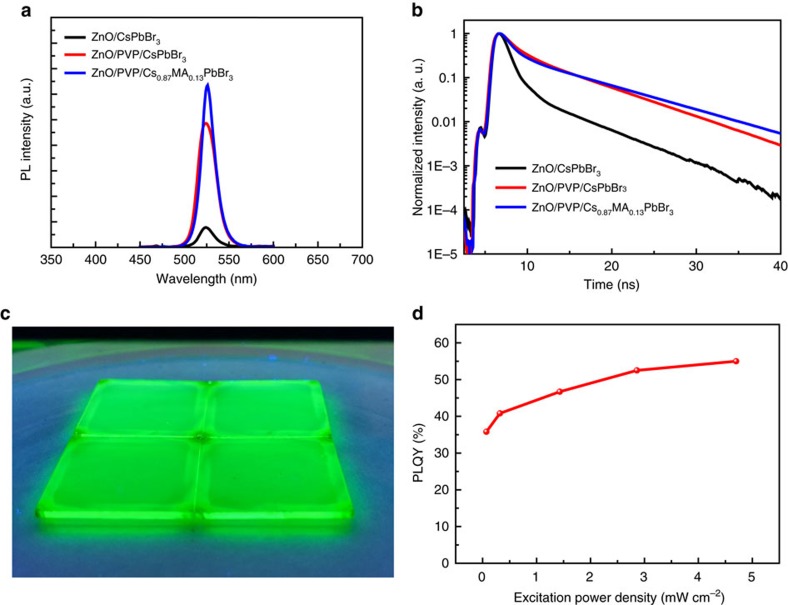
PL behaviour of CsPbBr_3_ films deposited under different conditions. (**a**) Steady-state PL of CsPbBr_3_ films on ZnO, ZnO/PVP and Cs_0.87_MA_0.13_PbBr_3_ film on ZnO/PVP, respectively, here PVP is polyvinyl pyrrolidine, MA is CH_3_NH_3_. (**b**) Time-resolved PL of CsPbBr_3_ films on ZnO and ZnO/PVP and Cs_0.87_MA_0.13_PbBr_3_ on ZnO/PVP. (**c**) PL image of Cs_0.87_MA_0.13_PbBr_3_ films on ZnO/PVP under ultraviolet lamp excitation. (**d**) PLQY of Cs_0.87_MA_0.13_PbBr_3_ as a function of excitation power density.

We carried out steady-state PL on CsPbBr_3_ thin films deposited from different conditions (Fig. 2a). The CsPbBr_3_ films directly deposited on ZnO showed weak green emission at 524 nm with a full width at half maximum (FWHM) of 24 nm. Perovskites deposited onto the PVP-modified ZnO showed a dramatic increase in PL intensity, indicating that non-radiative recombination in the perovskite layer or at the interfaces has been significantly suppressed. The enhancement of PL is posited to arise from several phenomena. First, the improved morphology may reduce non-radiative recombination at grain boundaries (Fig. 1a,b), leading to enhanced PL. Second, PVP could passivate ZnO surface defects^41^, which could act as non-radiative recombination traps at the interface of ZnO/perovskite. After PVP modification, the PL emission intensity and carrier lifetime of ZnO increased considerably (Supplementary Fig. 8), indicating a reduced surface defects of ZnO with PVP layer. Similar enhancement were also observed while coating PVP on perovskite surface, further confirming our argument (Supplementary Fig. 8).

The PL was further improved by introducing MABr into the CsPbBr_3_ lattice. This increase in PL intensity is consistent with the reduction of perovskite grain boundaries, as well as the suppression of Pb metallic recombination centres, which were confirmed by scanning electron microscopy (SEM) and XPS results, respectively (Fig. 1c and Supplementary Fig. 7). The emission peak from CsPbBr_3_ has been slightly shifted from 524 to 526 nm after adding MABr, indicating that a small fraction of MA cations have been introduced into the CsPbBr_3_ crystal lattice. A single Gaussian emission peak at 526 nm shows that the CsPbBr_3_ layer with MABr additive is a pure perovskite phase, which could be ascribed to the formation of an alloy phase of Cs_1*−x*_MA_*x*_PbBr_3_. We estimate that the MA content in this alloyed perovskite is 0.13, that is, Cs_0.87_MA_0.13_PbBr_3_-based on the band-edge emission as shown in Fig. 2a, and the band-edge emission form pure MAPbBr_3_ (540 nm)^28^ according to the linear relationship E_g,Cs1−*x*MA*x*PbBr3_=(1*−x*)E_g,CsPbBr_3__+*x*E_g,MAPbBr3_ (ref. 42). The final MABr content in CsPbBr_3_ as estimated from the change in bandgap is approximately consistent with the initial precursor composition where CsBr:PbBr_2_:MABr=2.2:1:0.1 and only 1 mol CsBr contributes to the formation of CsPbBr_3_ and the initial MABr ratio is 0.1 mol. The linear absorption of CsPbBr_3_ with and without MABr was consistent with the PL results (Supplementary Fig. 4). We further observed that the PL FWHM narrowed from 24 nm to 18 nm after introducing MABr. This indicates that the MABr additive has improved the sharpness of the perovskite band edge.

**Figure 3:**
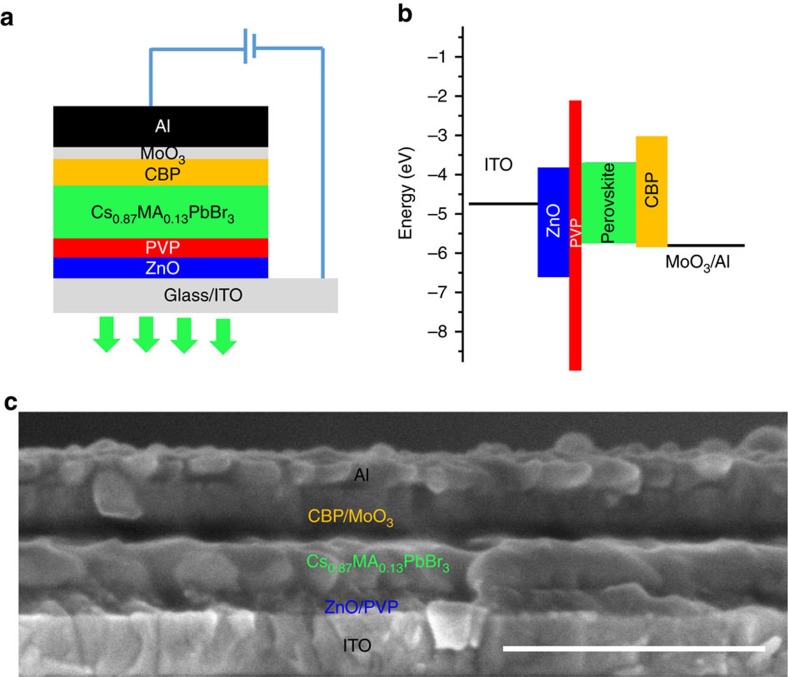
Device structure of CsPbBr_3_ inorganic-based perovskite LEDs. (**a**) Device structure, glass/ITO/ZnO/PVP/CsPbBr_3_/CBP/MoO_3_/Al, here PVP is polyvinyl pyrrolidine, CBP is 4,4′-Bis(N-carbazolyl)-1,1′-biphenyl. ZnO are CBP/MoO_3_ are used as the electron and hole injection layers, respectively. PVP was used to improve peorvskite morphology and also passivate the interface defects and improve charge injection balance. (**b**) Band alignment of each functional layer. (**c**) Cross-sectional SEM image of the LEDs, scale bar is 500 nm.

We next acquired time-resolved PL decay spectra of the different perovskite layers (Fig. 2b). The time-resolved PL curves were fit to bi-exponential decays, where the fast decay component is associated with trap-assisted recombination at grain boundaries or surfaces, and the slow decay is ascribed to radiative recombination inside the bulk perovskite phase^28^,^43^. For the ZnO/CsPbBr_3_, PVP/CsPbBr_3_ and PVP/Cs_0.87_MA_0.13_PbBr_3_ films, the decay times are (*τ*_1_=1.2 ns, *τ*_2_=4.6 ns), (*τ*_1_=2.1 ns, *τ*_2_=6.4 ns) and (*τ*_1_=1.8 ns, *τ*_2_=7.5 ns), respectively. Generally, it was found that the PL lifetime of the perovskite film is increased after PVP modification, and further increased after the addition of MABr. We observed that the Cs–MA mixed perovskite has a marginally faster decay component in comparison with pure Cs perovskite. We hypothesize that this may be a result of increased surface defects in the Cs–MA mixed perovskite. However, the slow decay component of Cs–MA exhibited a longer lifetime, indicative of less bulk defects, consistent with the observed reduction of Pb metallic states (Supplementary Fig. 7). Although Cs–MA sample showed shorter lifetime in fast decay component compared with pure Cs, stronger PL from Cs–MA samples (Fig. 3a) indicated that the overall defects including surface and bulk defects in Cs–MA are less than that of in pure Cs. The Cs_0.87_MA_0.13_PbBr_3_ films show bright and uniform green PL under ultraviolet lamp excitation (Fig. 2c). Both PVP interface engineering and MABr lattice incorporation enhanced the PL emission of the perovskite film, which is beneficial to realize high performance LEDs.

The PLQY of Cs_0.87_MA_0.13_PbBr_3_ was measured as a function of excitation power density (Fig. 2d). As seen in other perovskites, the PLQY increases with excitation power^30^,^31^. This is attributed to state-filling of recombination centres in the perovskite layer^30^,^31^. Our inorganic-based perovskite materials, Cs_0.87_MA_0.13_PbBr_3_, exhibited high quantum yield (35.8%) even at low light intensity (0.07 mW cm^−2^). This is significantly higher than previous reports at a similar order of power excitation, indicative of reduced non-radiative recombination centres in the perovskite layer^30^,^31^. Upon increasing the excitation intensity to 4.70 mW cm^−2^, the quantum efficiency increased to as high as 55%. The high PL quantum yield suggests promise for high EQE LEDs.

### Light-emitting diodes based on CsPbBr_3_ films

We fabricated LEDs consisting of glass/indium tin oxide (ITO)/ZnO/PVP/CsPbBr_3_/CBP/MoO_3_/Al (Fig. 3a), where CsPbBr_3_ is the emitting layer, Apart from ITO and MoO_3_/Al, which were deposited in vacuum, all layers were solution processed via spin coating. The band alignment of the CsPbBr_3_ LEDs could be drawn as shown in Fig. 3b based on the band structure of CsPbBr_3_ and ZnO (Supplementary Figs 3 and 5), and also the valence band of CBP (−6.0 eV)^39^. ZnO and CBP/MoO_3_ are used as the electron and hole injection layers, respectively. In addition to improve perovskite morphology and also passivate the interface defects, which has been illustrate above (Figs 1 and 2). PVP layer could also induce an electron-injection barrier (Fig. 3b), which could improve charge injection balance, this will be discussed later. The electrons and holes injected from each side recombine radiatively in the perovskite layer, resulting in photon emission. A cross-sectional SEM image of a typical device showed a clear sandwich structure (Fig. 3c). The thicknesses of the ZnO/PVP, CsPbBr_3_ and 4,4′-Bis(N-carbazolyl)-1,1′-biphenyl (CBP)/MoO_3_ layers are ∼45 nm, 100 nm and 80 nm, respectively.

### Electroluminescence of light-emitting diodes

**Figure 4:**
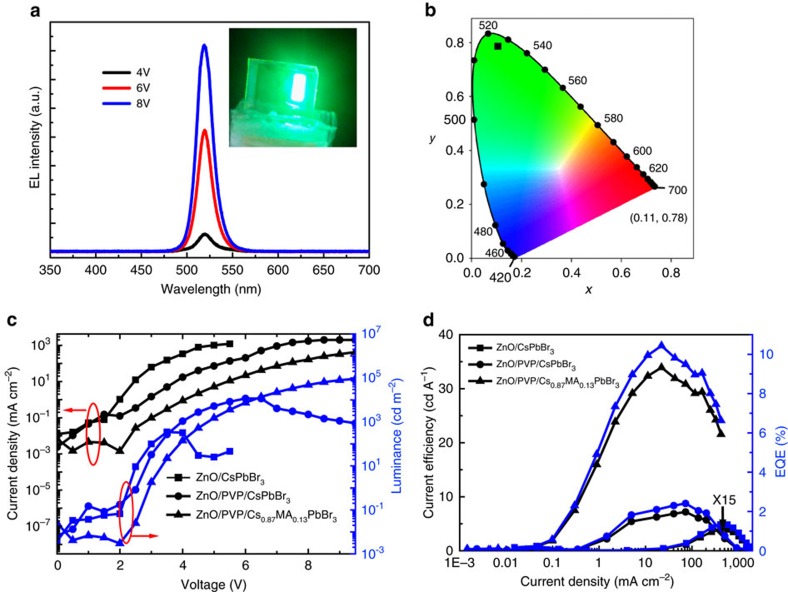
EL performance of the devices. (**a**) EL spectra of Cs_0.87_MA_0.13_PbBr_3_-based devices under varying voltage bias (the emission image is shown in inset). (**b**) The corresponding CIE coordinate. (**c**) *I*–*V* and voltage–light intensity (*L*–*V*) curves for the devices with and without PVP buffer layer or with and without CH_3_NH_3_Br (MABr) additive, that is, ZnO/CsPbBr_3_, ZnO/PVP/CsPbBr_3_ and ZnO/PVP/Cs_0.87_MA_0.13_PbBr_3_, respectively, here PVP is polyvinyl pyrrolidine. (**d**) Current efficiency and EQE of devices with and without PVP buffer layer, with and without CH_3_NH_3_Br (MABr) additive, that is, ZnO/CsPbBr_3_, ZnO/PVP/CsPbBr_3_ and ZnO/PVP/Cs_0.87_MA_0.13_PbBr_3_, respectively.

The EL spectra of CsPbBr_3_ and Cs_0.87_MA_0.13_PbBr_3_-based devices are centred at 516 and 520 nm, respectively (Supplementary Fig. 9). Compared to the PL emission at 526 nm for Cs_0.87_MA_0.13_PbBr_3_, the EL emission showed a slight blue shift to 520 nm, which has also been observed in other perovskite-based LEDs^16^,^44^. The blue shift in the EL spectrum could be ascribed to free carrier emission, as already demonstrated in several perovskite systems^16^,^44^,^45^. For the devices using Cs_0.87_MA_0.13_PbBr_3_ as an emitting layer, the EL spectrum as a function of voltage bias was measured (Fig. 4a), and an EL image of the device under operation was taken (inset of Fig. 4a). The EL showed very narrow emission (FWHM=18 nm) and high colour purity. This spectral line width is narrower than that of previously reported perovskite nanocrystal-based LEDs^14^,^15^. The devices exhibited saturated and pure colour (90.2%) at green wavelengths, with Commission Internationale de l'Eclairage (CIE) chromaticity coordinates at (0.11, 0.78) (Fig. 4b).

We measured the voltage–current (*I–V*) curve of the devices (Fig. 4c) and found that the control devices (without both PVP and MABr) showed higher injection current. This could be two reasons: one is high density of pinholes in the perovskite layer which results in a significant electron and hole injection leakage; and another could be the imbalanced charge injection. After introducing the PVP buffer layer and the MABr additive, the injection current of CsPbBr_3_ device was significantly reduced, indicating that the current leakage and charge injection imbalance has been suppressed. The turn-on voltage was slightly increased after inserting an immediate layer of PVP, which could be mainly due to the injection barrier caused by the insulating nature of PVP layer^46^. The further minor increase of turn-on voltage after MABr incorporation might be ascribed to deeper valence band of Cs_0.87_MA_0.13_PbBr_3_ (5.71 eV) compared to CsPbBr_3_ (5.50 eV) (Supplementary Fig. 3, Supplementary Table 1)^47^. Similar phenomenon has also been found by Sun *et al*. in FA–Cs mixture nanocrystal-based LEDs^48^. Although the turn-on voltage increased after incorporating PVP buffer layer and adding MABr salt, it can be calculated that the current efficiency are significantly increased from 0.02 cd A^−1^ (CsPbBr_3_) to 1 cd A^−1^ (PVP/Cs_0.87_MA_0.13_PbBr_3_) at small brightness (1 cd m^−2^). The increase of current efficiency indicated the non-radiative recombination has been suppressed, which will be discussed later. The control devices showed a maximum brightness of 300 cd m^−2^ (Fig. 4c). The maximum brightness was dramatically increased to 11600, cd m^−2^ by introducing the PVP intermediate layer. Consistent with this was an observed increase in the current efficiency from 0.26 cd A^−1^ to 7.19 cd A^−1^ (Fig. 4d). The EQE of the LEDs from control devices of CsPbBr_3_ on ZnO is <0.1%. The addition of the PVP buffer layer improved the quantum efficiency to 2.4% (Fig. 4d).

The MABr salt additive further improves both the maximum brightness and EQE. The maximum brightness of Cs_0.87_MA_0.13_PbBr_3_-based LEDs increased to as high as 91,000 cd m^−2^. The current efficiency and quantum efficiency were increased to 33.9 cd A^−1^ and 10.43%, respectively (Fig. 4d). The best devices exhibited an internal quantum efficiency (IQE) of 47% calculated by IQE=2*n*^2^EQE, where n is the refractive of glass (1.5)^31^. The device performance parameters are summarized in Table 1. To the best of our best knowledge, these devices are the brightest and most-efficient perovskite-based LEDs emitting at green wavelengths reported to date.

These devices also demonstrated high reproducibility (Supplementary Fig. 10). The high performance in the ZnO/PVP/Cs_0.87_MA_0.13_PbBr_3_ LED is a result of careful optimization of the interfaces and perovskite layers (Supplementary Figs 11–13, Supplementary Tables 2 and 3). It was found that a high amount of MABr compositionally mixed with CsPbBr_3_ led to a significant decrease in LED performance. We attribute this to poor morphology (Supplementary Fig. 14).

We tested the stability of CsPbBr_3_ LEDs (Supplementary Fig. 15). Consistent with what was observed in previous perovskite LED reports^33^,^49^, the devices decayed after several minutes. The decay mechanism is hypothesized to be a result of ion migration under steady-state voltage bias. To improve device stability, we attempted to incorporate ion-migration-inhibiting polymers^50^, such as PVP, into the perovskite layer. The results indeed showed that the stability had been significantly improved—the output was stable for several hours. While encouraging, the efficiency of this polymer-blended device is reduced in comparison with the ZnO/PVP/Cs_0.87_MA_0.13_PbBr_3_ devices (Supplementary Fig. 16). We will study in more depth the underlying phenomena leading to this stability/efficiency compromise in our future work. We have also tested the transient light emission response of our perovskite LEDs (Supplementary Fig. 17). Nearly instantaneous turn-on was achieved with a response time about 18 ms to reach their maximum output light intensities. Such a fast turn-on is comparable to that of conventional LEDs^51^.

## Discussion

We found that the improvement via PVP could be due to three reasons. First, the improvement of the perovskite film morphology, as shown in Fig. 1a,b, leads to reduced pinholes which minimize current leakage (Fig. 4a). Second, the suppression of non-radiative recombination at the ZnO/perovskite interface, which has been confirmed by PL results (Fig. 2a, Supplementary Fig. 8), improves the radiative efficiency.

In addition to improvement of perovskite film morphology and passivation of defects at interface, PVP could improve the charge injection balance in our perovskite LEDs, and thus enhancing devices EL efficiency. Similar mechanism has been proposed in previous reports, while using PMMA insulting layer in quantum dot LEDs^46^. It was found that the injection current from electron only devices is much higher than that of the current from hole only devices, while using ZnO and CBP as the main injection layers, respectively (Supplementary Fig. 18). These results indicated that the electron injected by ZnO is faster than holes injected by CBP, which could be due to the different carrier mobility of ZnO and CBP (ref. 46). This will lead to charge injection imbalance and also the excess electron current while using these layer as electron- and hole-injection layers in LEDs, and thus degrading EL efficiency^46^. Insulting PVP layer can slow down electron-injection via an energy barrier (Fig. 3b, Supplementary Fig. 18), an improvement of charge balance could be anticipated by inserting a PVP layer on ZnO surface as the electron-injection layer. In fact, the reduction of device injection current via PVP insertion confirmed the improvement of charge injection balance (Fig. 4c)^46^, and thus improving EL emission efficiency.

There could be two key improvements of leading to superior performance for MA–Cs mixed perovskite devices. The first one is ascribed to the suppression of non-radiative recombination centres by eliminating the Pb metallic phase by compositionally blending CsPbBr_3_ with MABr to form the compound Cs_0.87_MA_0.13_PbBr_3_. PL (Fig. 2a) and time-resolved PL (Fig. 2b) both indicated that the less defects in Cs–MA films. Second, the key advance that led to the dramatic improvement in EL brightness and efficiency is the reduction of leakage current via improved morphology as a result of both PVP-modified ZnO and the MABr additive (Fig. 1c).

In summary, we have obtained high-quality Cs_0.87_MA_0.13_PbBr_3_ perovskite light-emitting thin films with minimized pinholes through electron-injecting interface passivation and perovskite composition modulation. These strategies jointly reduced the device leakage current. Furthermore, the non-radiative recombination centres at the interfaces and in the perovskite film were suppressed and also the charge injection balance were improved. As a result of these advances, we obtained ultra-bright and highly efficient inorganic perovskite-based LEDs. With additional optimizations to the perovskite and interfacial layers, the inorganic perovskite-based LEDs have promise to reach 20% EQE, making them competitive with materials such as semiconducting organics and colloidal quantum dots.

## Methods

### Preparation of perovskite solution and ZnO nanoparticles

CsBr (Sigma Aldrich, 99.9%) and PbBr_2_ (Aldrich, 99.99%) (CsBr:PbBr_2_ molar ratio of 2.2:1) solutions were prepared using DMSO as a solvent. The solution concentration is 0.5 M (CsBr 1.1 M, PbBr_2_ 0.5 M). A high ratio of CsBr:PbBr_2_ was used to suppress the formation of non-CsPbBr_3_ phases. For films which incorporated the CH_3_NH_3_Br additive, 0.05 M CH_3_NH_3_Br was added to the solution (CsBr:PbBr_2_:MABr=2.2:1:0.1). Comparative studies used additional ratios of CsPbBr_3_ solutions, such as CsBr:PbBr_2_:MABr=2:1:0.1, 2.1:1:0.1, 2.2:1:0.1, 2.4:1:0.1 and 2.2:1:0.2. The precursor solutions were stirred at 45 °C overnight. And then the solution was stand for 4 h at room temperature, precipitates were formed in the CsBr-rich solution, top transparent solution was decanted and filtered for using. The details of precursor preparation procedures were shown in Supplementary Fig. 19. The ZnO nanoparticles were synthesized using a previously developed method^8^. The synthesized ZnO nanoparticles were dispersed in methanol and n-butanol to form a 2% ZnO nanoparticle solution.

### Light-emitting diode fabrication

The glass/ITO substrate was sequentially washed with isopropanol, acetone, distilled water and isopropanol. The sheet resistance of ITO is 15 Ω per square. ZnO nanoparticles of concentration 2% by weight were spin-coated onto ITO substrates at 2,000 r.p.m. for 30 s and then annealed at 150 °C for 15 min. For control devices, perovskite precursor (CsBr:PbBr_2_=2.2:1) was spin-coated onto ZnO at 2,000 r.p.m. for 2 min, and then annealed at 100 for 20 min. After, a 2wt% CBP solution was spin-coated onto the perovskite. The devices were transferred into a vacuum chamber for MoO_3_/Al deposition. For PVP interface-modified devices, 0.5 wt% PVP solution in DMSO was spin-coated onto ZnO at 2,000 r.p.m. for 1 min, and then annealed at 150 ^°^C for 15 min to induce crosslinking. However, we found that the PVP is slightly washed away during spin-coating of the DMSO solution. The PVP thickness before and after DMSO solution washing are 8.4 and 5.0 nm, respectively, which were measured by ellipsometer (Supplementary Fig. 20). For MABr additive devices, the ratio of CsBr:PbBr_2_:MABr is 2.2:1:0.1, the final composition is Cs_0.87_MA_0.13_PbBr_3_ as determined by band-edge emission measurements. The solution concentration was 0.5 M. The device active area was 0.108 cm^2^.

### Materials and device characterization

A field emission SEM (FEI NanoSEM650) was used to acquire SEM images. The instrument uses an electron beam accelerated at 500 V to 30 kV, enabling operation at a variety of currents. Absorption measurement were carried out by Hitachi ultraviolet–visible U-4100 spectrophotometer. Absorbance was determined from a transmittance measurement using integrated sphere. PL measurements were carried out by FLS980 Spectrometer. The X-ray diffraction patterns (*θ*–2*θ* scans) were taken on a Rigaku D/MAX-2500 system using Cu kα (*λ*=1.5405 Å) as the X-ray source. Scans were taken with 0.5 mm wide source and detector slits, and X-ray generator settings at 40 kV and 30 mA. XPS was performed on the Thermo Scientific ESCALab 250Xi using 200 W monochromated Al Kα (1,486.6 eV) radiation. The 500 μm X-ray spot was used for XPS analysis. The base pressure in the analysis chamber was ∼3 × 10^−10^ mbar. Typically the hydrocarbon C1s line at 284.8 eV from adventitious carbon is used for energy referencing. UPS samples were analyzed on a Thermo Scientific ESCALab 250Xi. The gas discharge lamp was used for UPS, with helium gas admitted and the HeI (21.22 eV) emission line employed. The helium pressure in the chamber during analysis was ∼2E−8 mbar. The data was acquired with a −10 V bias. The work function of the measured sample can be calculated from following equation: *hν*−*φ*=*E*_Fermi_−*E*_cutoff_, here, *hν*=21.22 eV, *E*_Fermi_=21.08 eV (using Ni as the standard sample for calibration), *E*_cut-off_ is the cut-off shown in the corresponding Figures. The PLQY was measured using a Horiba Fluorolog system equipped with a single grating and a Quanta-Phil integration sphere coupled to the Fluorolog system with optic fibre bundles^30^. The following settings were applied for PLQY measurements: an excitation wavelength of 400 nm; bandpass values of 10 and 5 nm for the excitation and emission slits, respectively; step increments of 1 nm and integration time of 0.5 s per data point. The excitation power density in the power-dependent PLQY characterization was tuned by varying the slit width on the Fluorolog monochromator. *J–V* characteristics of LEDs were taken using a Keithley 2,400 source metre. Two Keithley 2,400 source metre units linked to a calibrated silicon photodiode were used to measure the current–voltage–brightness characteristics. The measurement system has been carefully calibrated by efficient InGaN/GaN LEDs with a similar photon response by PR-650 SpectraScan. A Lambertian profile was assumed in the calculation of EQE^28^,^30^,^31^.

### Data availability

The data that support the findings of this study are available from the corresponding author upon reasonable request.

## Additional information

**How to cite this article:** Zhang, L. *et al*. Ultra-bright and highly efficient inorganic based perovskite light-emitting diodes. *Nat. Commun.* **8**, 15640 doi: 10.1038/ncomms15640 (2017).

**Publisher's note:** Springer Nature remains neutral with regard to jurisdictional claims in published maps and institutional affiliations.

## Supplementary Material

Supplementary Information Supplementary Figures and Supplementary Tables

## Figures and Tables

**Table 1:** Device performance with and without PVP intermediate layers or CH_3_NH_3_Br (MABr) additive.

**Devices**	***V***_**th**_**(V)**	***L***_**max**_**(cd m**^**−2**^**)**	**Current efficiency(Cd A**^**−1**^**)**	**EQE (%)**
ZnO/CsPbBr_3_	2.3	350	0.26	0.09
ZnO/PVP/CsPbBr_3_	2.6	11,600	7.19	2.41
ZnO/PVP/Cs_0.87_MA_0.13_PbBr_3_	2.9	91,000	33.9	10.43
