# Supplementary material for: Ultra-bright and highly efficient inorganic based perovskite light-emitting diodes
Source: Nat Commun. 2017 Jun 7;8:15640. doi: 10.1038/ncomms15640 (PMC5467226; doi:10.1038/ncomms15640)
Supplement: Supplementary Information — Supplementary Figures and Supplementary Tables [file ncomms15640-s1.pdf]

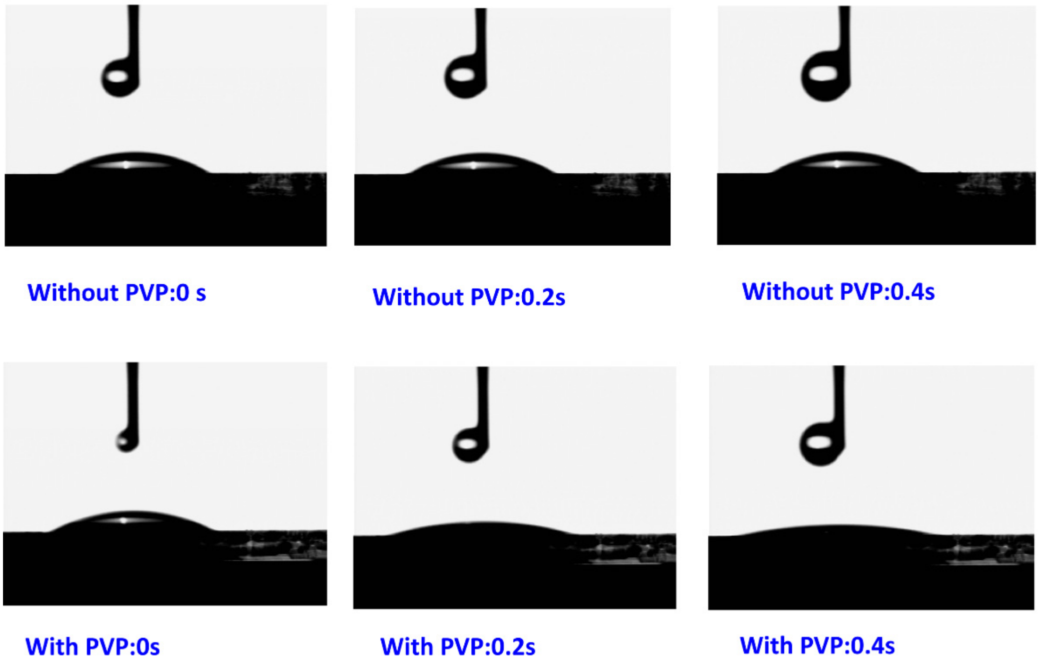

Supplementary Figure 1| Real time contact angle measurements for H_2O deposited onto ZnO and ZnO/PVP surfaces.

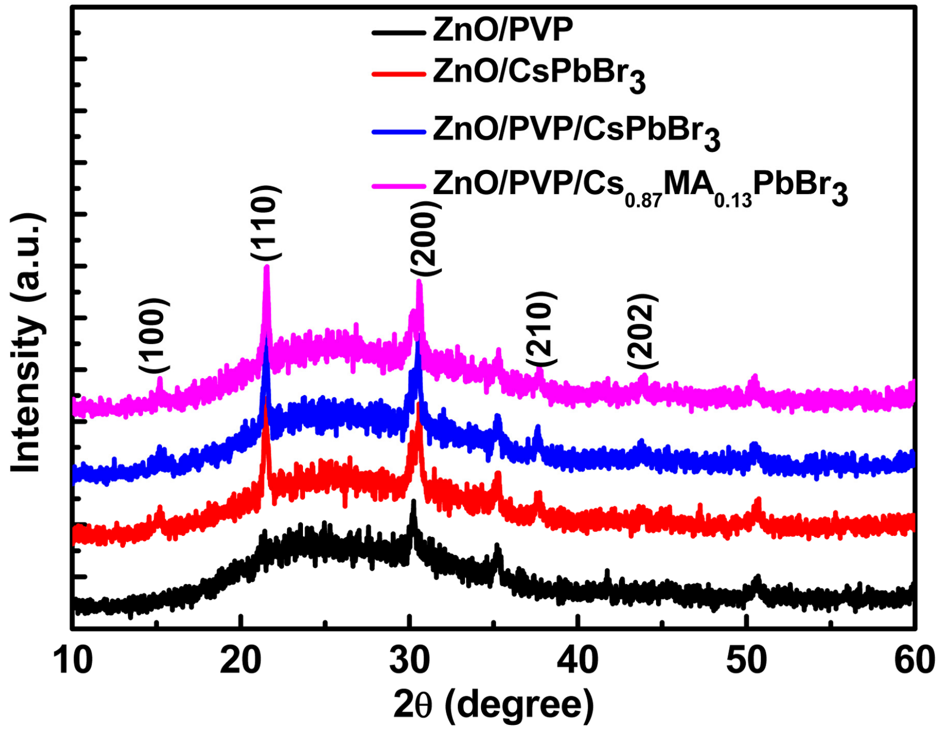

Supplementary Figure 2| X-ray diffraction (XRD) pattern of $CsPbBr_3$ on different substrates and with or without the CH_3NH_3Br (MABr) additive.

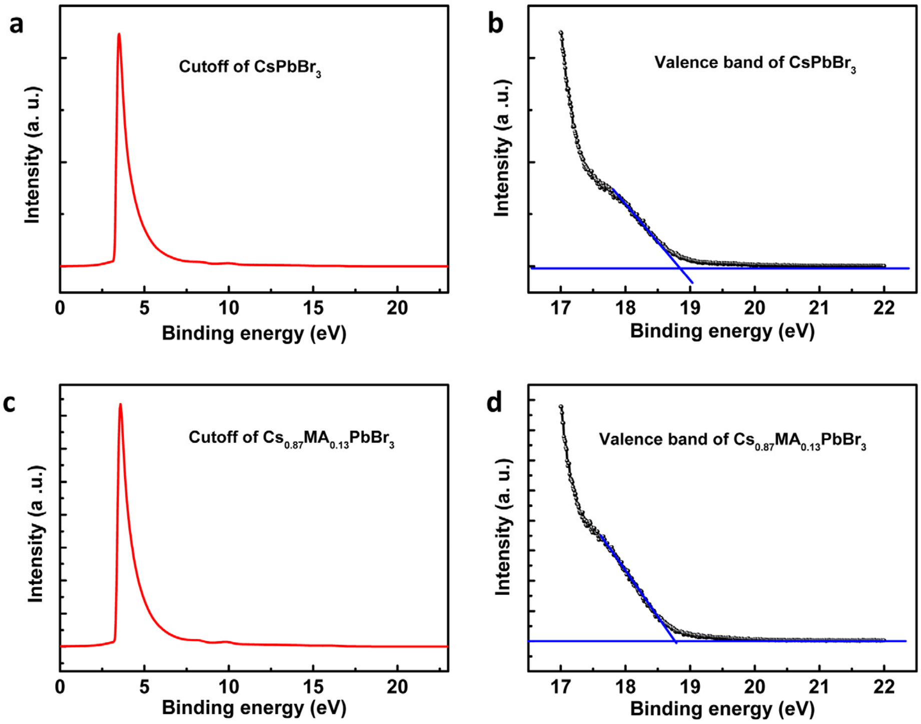

Supplementary Figure 3| Ultraviolet photoelectron spectroscopy (UPS) of $CsPbBr_3$ and $Cs_{0.87}MA_{0.13}PbBr_3$. The work function (W_F) was obtained from hν-W_F = E_{Fermi}-E_{cutoff}, here, hν = 21.22eV, E_{Fermi} = 21.08eV (using Ni as the standard sample for calibration), E_{cutoff} of $CsPbBr_3$ and $Cs_{0.87}MA_{0.13}PbBr_3$ are 3.22 eV, 3.30 eV respectively, from (**a**) and (**c**). Their work functions are 3.36 eV and 3.44 eV, respectively. (**b**) and (**d**) Perovskite valence band spectra with 21.08 eV defined as the Fermi level. The leading edges of the valence band spectra recorded from $CsPbBr_3$ and $Cs_{0.87}MA_{0.13}PbBr_3$ are 18.94 eV and 18.81 eV respectively. The valence band maximum (VBM) of these two materials are 2.14 eV and 2.27 eV respectively. From supplementary Figure 4, the bandgaps of $CsPbBr_3$ and $Cs_{0.87}MA_{0.13}PbBr_3$ are 2.35 eV and 2.34 eV, respectively. And the valence band E_V (E_V=W_F+VBM) of $CsPbBr_3$ and $Cs_{0.87}MA_{0.13}PbBr_3$ are 5.50 eV and 5.71 eV, respectively. The calculated conduction band energies E_C (E_C=W_F+VBM-Eg) of are 3.15 eV and 3.37 eV, respectively.

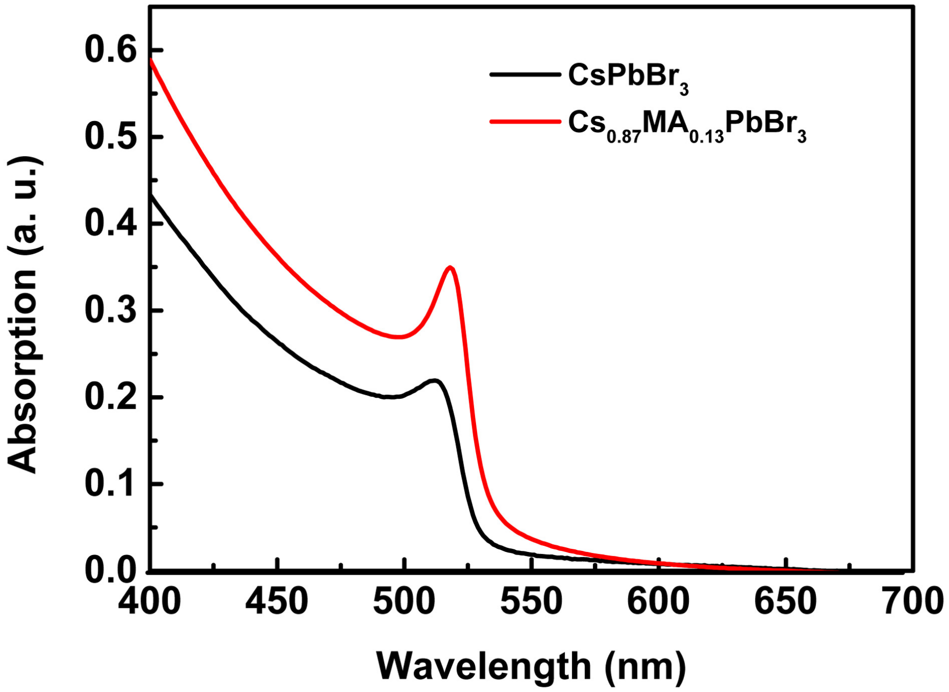

Supplementary Figure 4| Absorption of $CsPbBr_3$ and $Cs_{0.87}MA_{0.13}PbBr_3$. The absorption edges are located at 528 nm and 531 nm for $CsPbBr_3$ and $Cs_{0.87}MA_{0.13}PbBr_3$, respectively. Correspondingly, the bandgap of $CsPbBr_3$ and $Cs_{0.87}MA_{0.13}PbBr_3$ are 2.35 eV and 2.34 eV, respectively, based on the absorption edge.

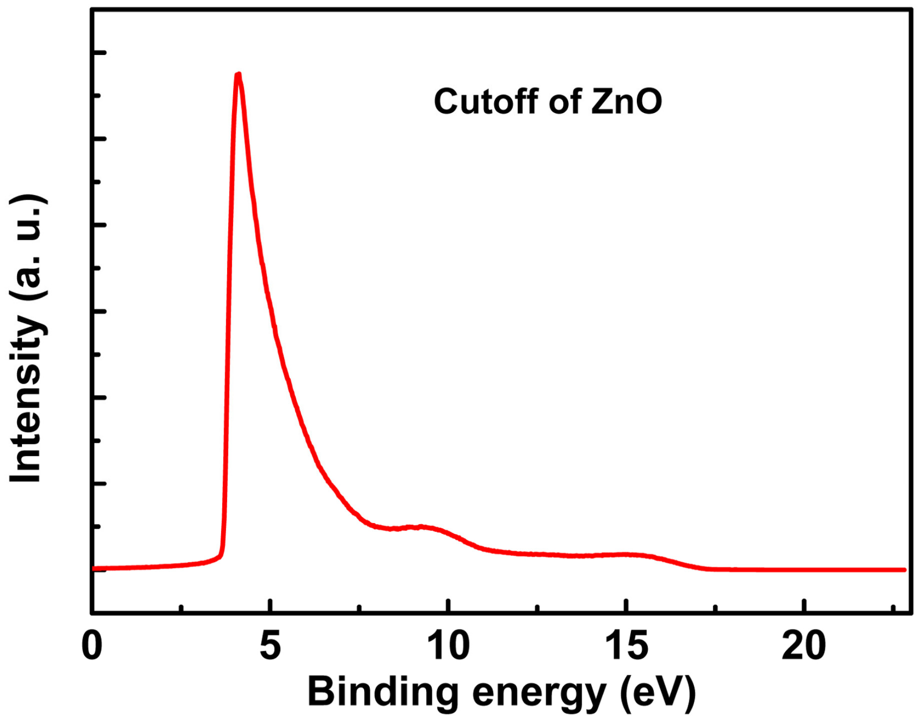

Supplementary Figure 5| Ultraviolet photoelectron spectroscopy (UPS) cutoff edge of thin film ZnO deposited onto ITO substrates. The cutoff of ZnO is 3.70 eV. According to the equation shown in supplementary Figure 3, the work function of ZnO is 3.84 eV.

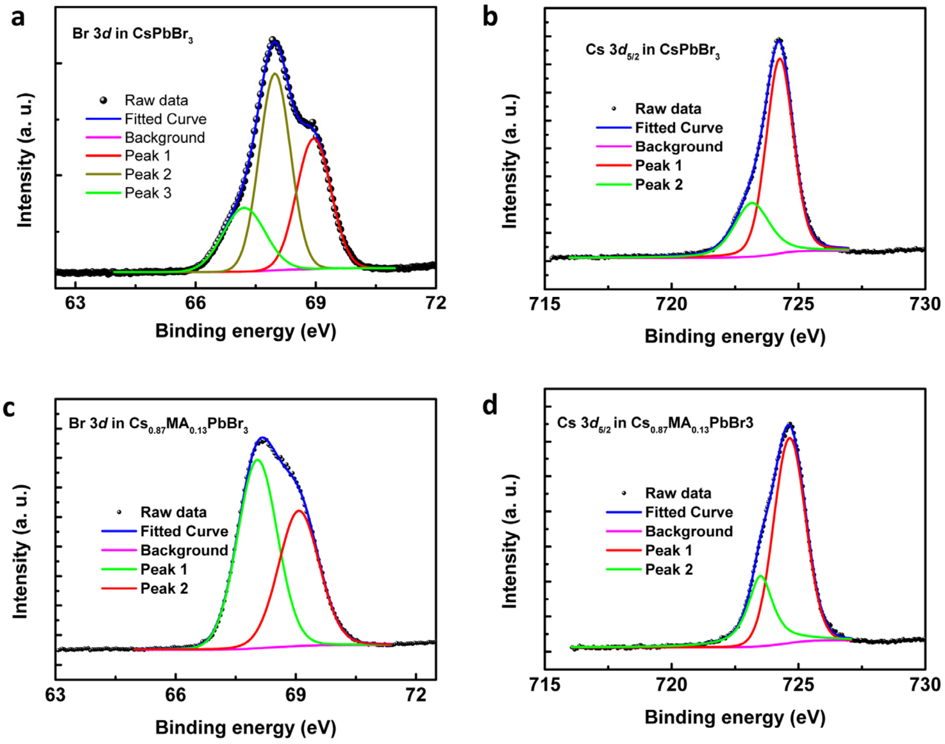

Supplementary Figure 6|. X-ray photoelectron spectroscopy (XPS) of $CsPbBr_3$ and $Cs_{0.87}MA_{0.13}PbBr_3$. **a**, Br 3*d* in $CsPbBr_3$, **b**, Cs 3$d_{5/2}$ in $CsPbBr_3$, **c,** Br 3*d* in $Cs_{0.87}MA_{0.13}PbBr_3$ and **d**, Cs 3$d_{5/2}$ in $Cs_{0.87}MA_{0.13}PbBr_3$.

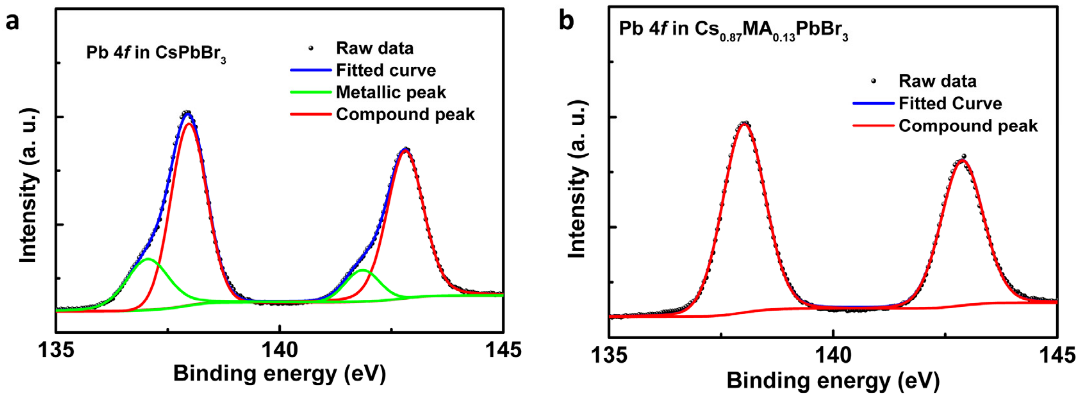

Supplementary Figure 7| X-ray photoelectron spectroscopy (XPS) of Pb 4*f* core level of $CsPbBr_3$ and $Cs_{0.87}MA_{0.13}PbBr_3$, respectively.

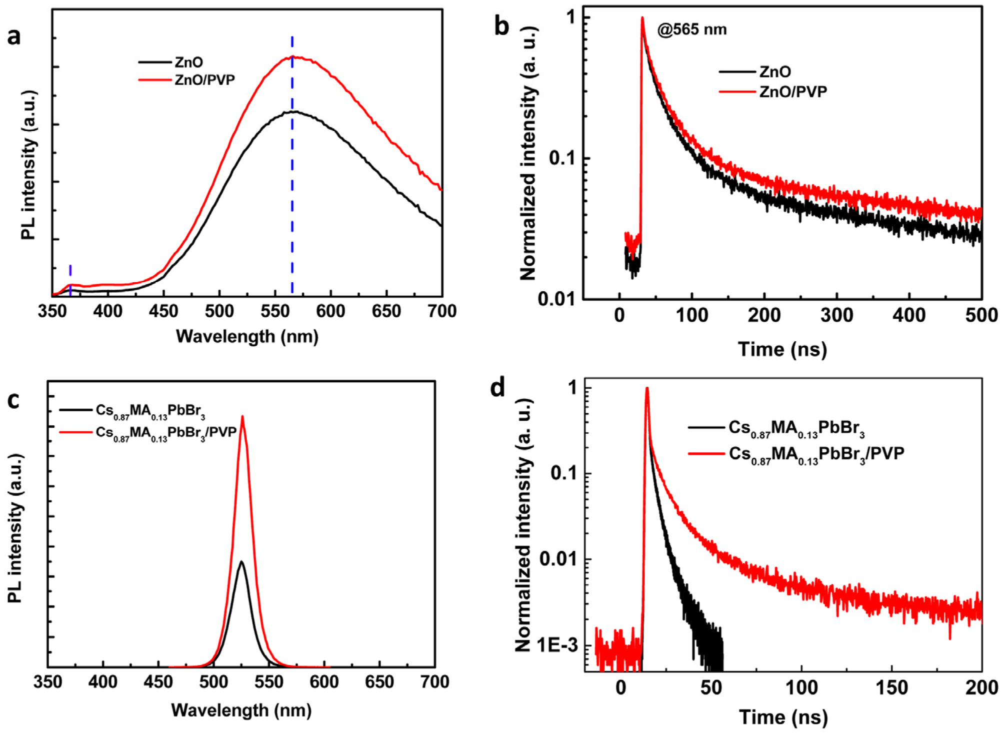

Supplementary Figure 8| Steady photoluminescence (PL) and time resolved photoluminescence (TRPL) of ZnO and perovskite before and after PVP coating. **a,** PL of ZnO before and after PVP coating. **b,** TRPL of ZnO before and after PVP coating, 565 nm emission was selected for TRPL measurement. **c,** PL of $Cs_{0.87}MA_{0.13}PbBr_3$ before and after PVP coating. **d**, TRPL of $Cs_{0.87}MA_{0.13}PbBr_3$ before and after PVP coating. All the measurement, the light excitation and emission collection were both from glass side.

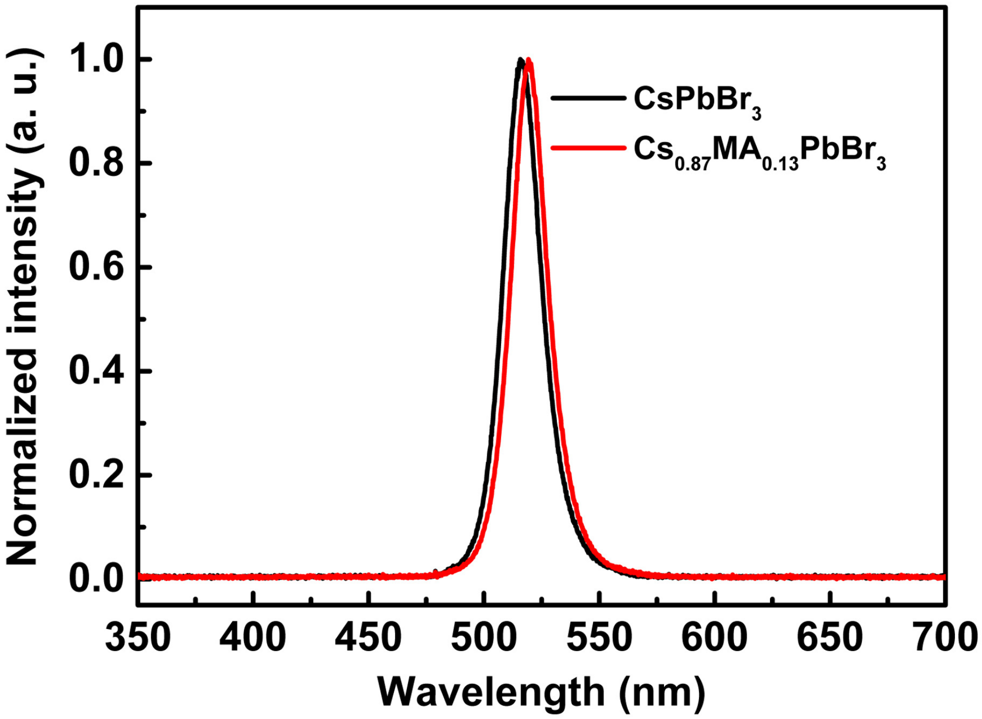

Supplementary Figure 9| Normalized electroluminescence (EL) from $CsPbBr_3$ and $Cs_{0.87}MA_{0.13}PbBr_3$ based light-emitting diodes.

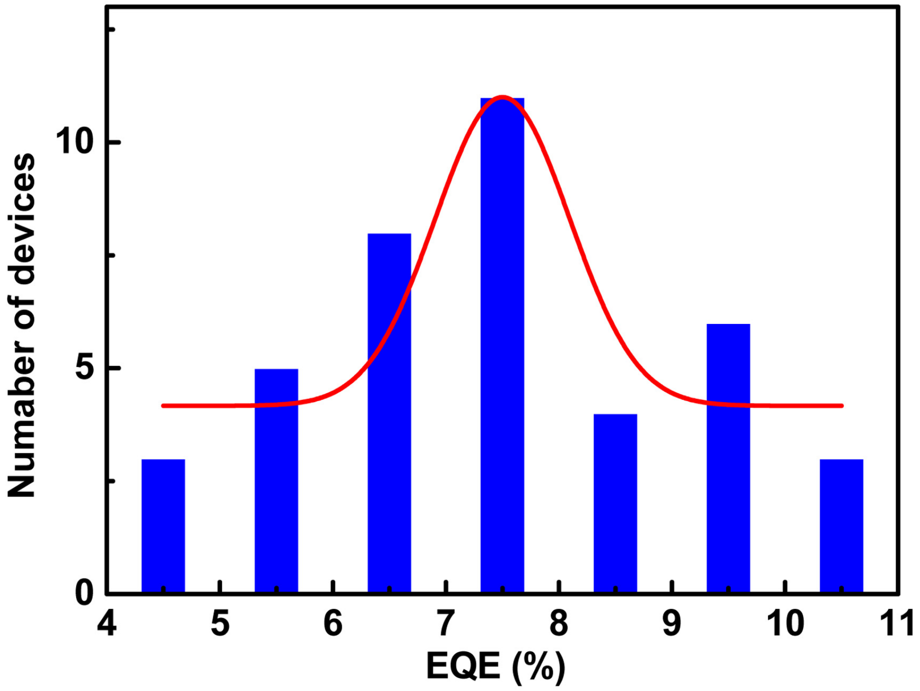

Supplementary Figure 10| External quantum efficiency (EQE) distribution from 50 devices using $Cs_{0.87}MA_{0.13}PbBr_3$ as the emitting layer.

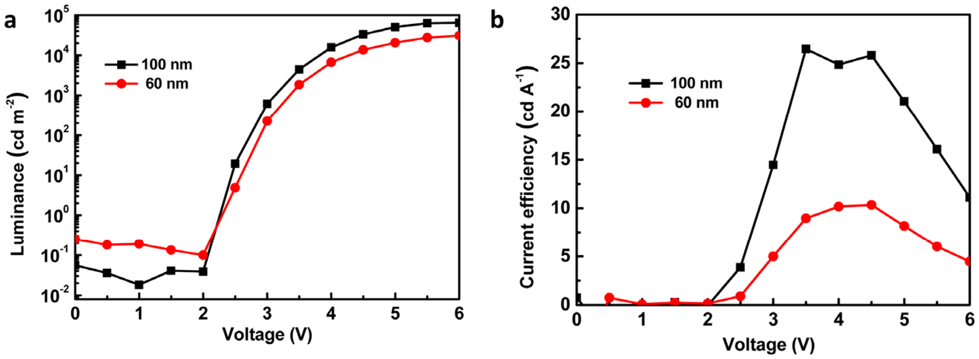

Supplementary Figure 11| Device performance with different thicknesses of the $Cs_{0.87}MA_{0.13}PbBr_3$ layer. **a**, L-V curve for the devices with 100 nm and 60 nm emitting layers. **b,** Current efficiency for the devices with 100 nm and 60 nm emitting layers.

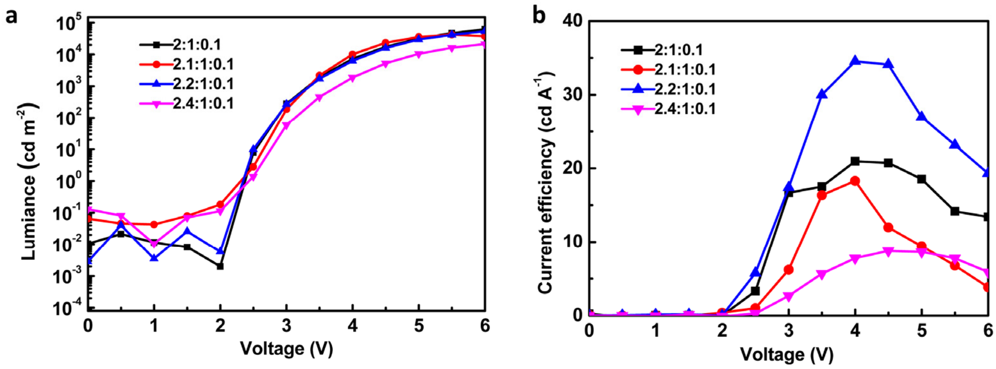

Supplementary Figure 12| Device performance with different ratio of CsBr in perovksite precursor solutions. **a,** L-V curve for the devices with different ratio of CsBr, CsBr:$PbBr_2$:MABr=x:1:0.1 (x=2, 2.1, 2.2, 2.4). **b,** Current efficiency for the devices with different ratios of CsBr, CsBr:$PbBr_2$:MABr=x:1:0.1 (x=2, 2.1, 2.2, 2.4).

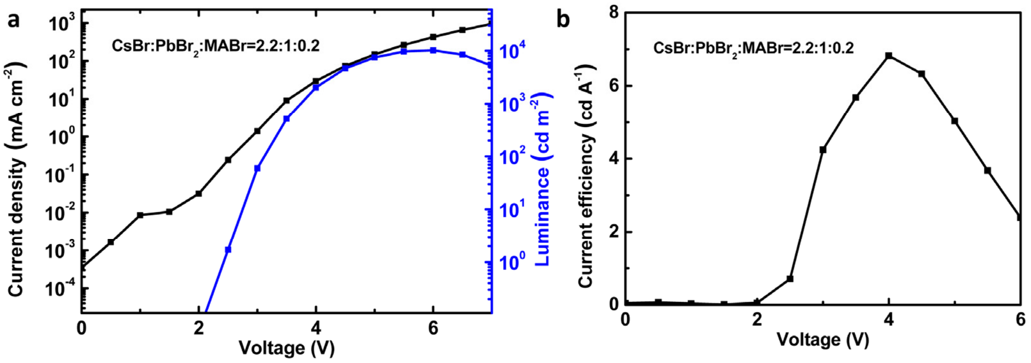

Supplementary Figure 13| Device performance with CsBr:$PbBr_2$:MABr=2.2:1:0.2. **a**, I-V curve and L-V curve. **b**, Current efficiency of the devices. It was found that the excess MABr additive (CsBr:$PbBr_2$:MABr=2.2:1:0.2) led to a decrease in device performance. The device showed a current efficiency of 6.82 cd A^{-1}.

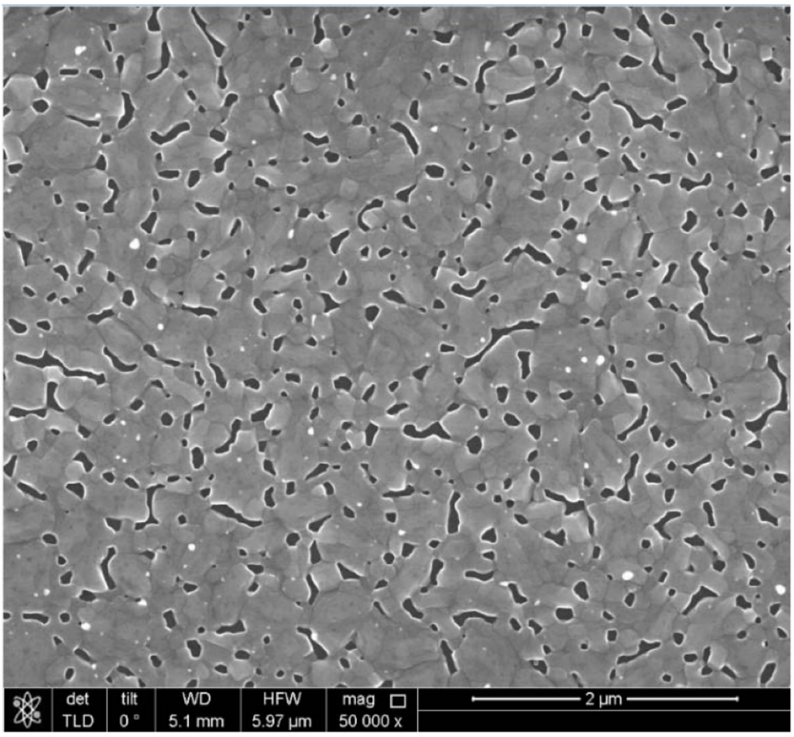

Supplementary Figure S14| Scanning electron microscopy (SEM) image of $Cs_{1-x}MA_xPbBr_3$ using $CsBr:PbBr_2:MABr$ with the ratio of 2.2:1:0.2. The x was assumed to be 0.26 based on the initial precursor composition and the final product of 2.2:1:0.1 (x=0.13).

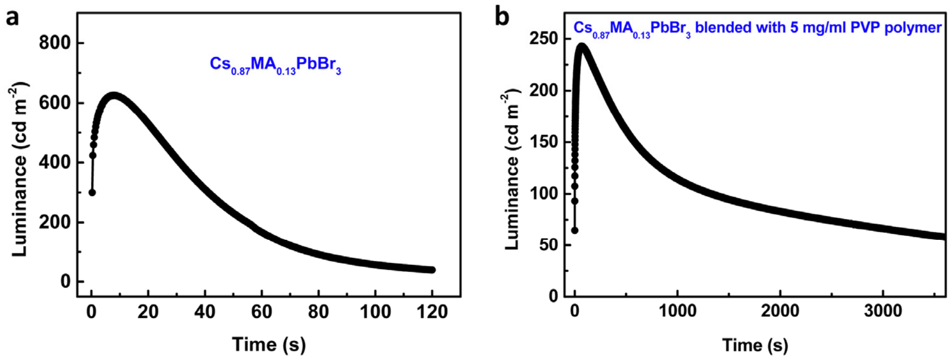

Supplementary Figure 15| Stability of the LEDs. **a**, stability of $Cs_{0.87}MA_{0.13}PbBr_3$ based LEDs under 3.7 V forward bias. **b**, stability of $Cs_{0.87}MA_{0.13}PbBr_3$ blended with PVP polymer under 5 V forward bias.

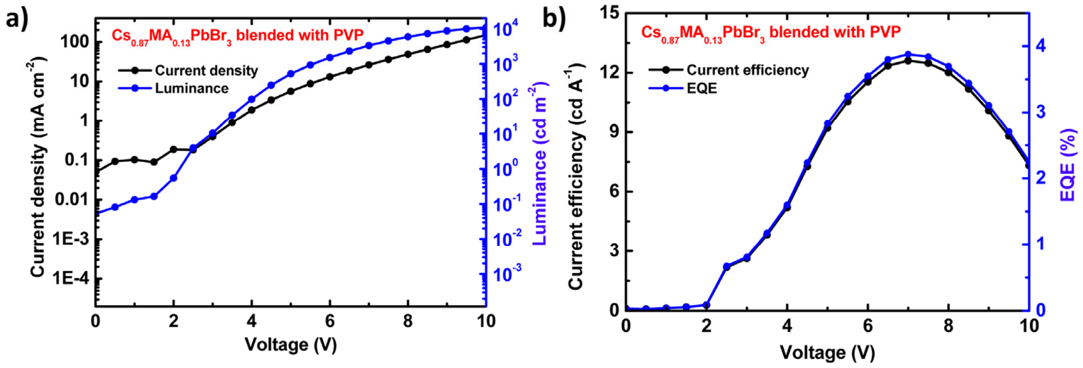

Supplementary Fig. 16| Device performance with CsBr:$PbBr_2$:MABr=2.2:1:0.1 blended with 5 mg/ml of PVP. **a**, I-V curve and L-V curve. **b**, Current efficiency and external quantum efficiency of the devices. The insulating PVP polymer mixed into the perovskite led to inefficient charge injection, low current efficiency, and low external quantum efficiency.

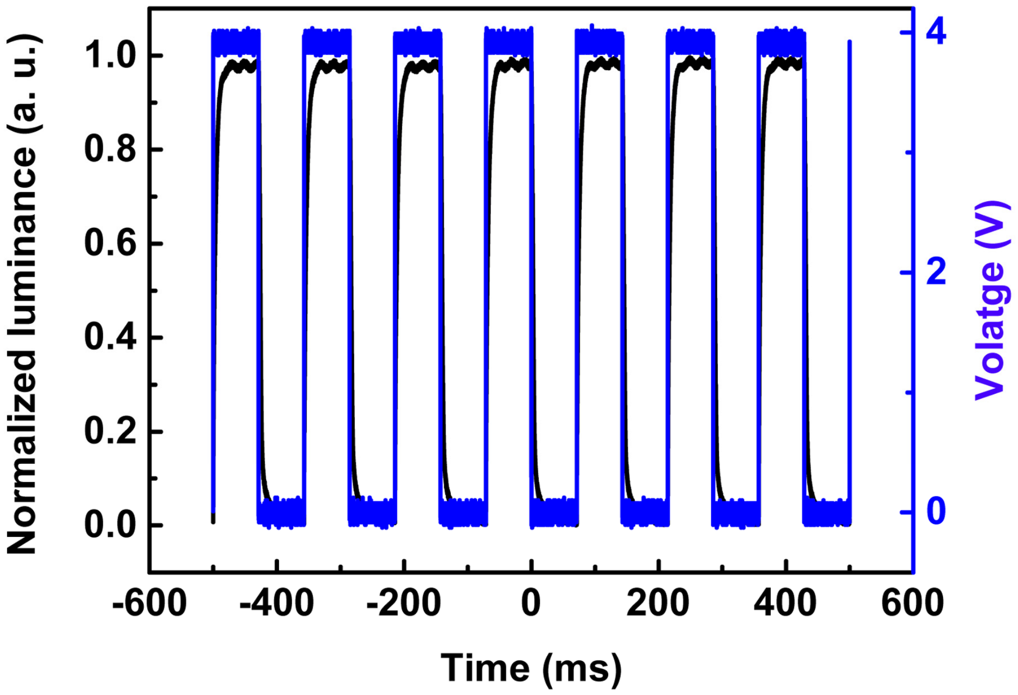

Supplementary Fig. 17| Transient light emission response under a 0−4 V pulse train at 7 Hz frequency.

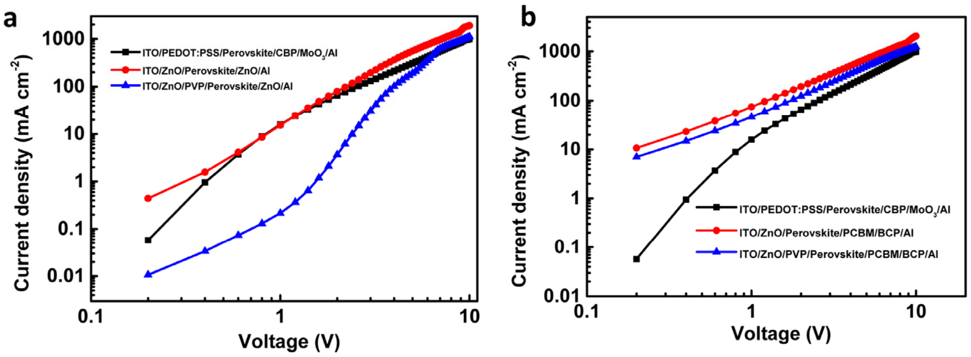

Supplementary Figure 18| The Electrical measurements on the electron-only devices (ITO/ZnO(PVP)/perovskite/ZnO/Al or ITO/ZnO(PVP)/perovskite/PCBM/BCP/Al) and the hole-only devices (ITO/PEDOT:PSS/perovskite/CBP/MoO_3/Al). From **a** and **b**, it can be found that the electron injection rate is much higher than that of hole injection rate, while inserting PVP, the electron injection rate can be slowed down, which could be better for charge injection balance.

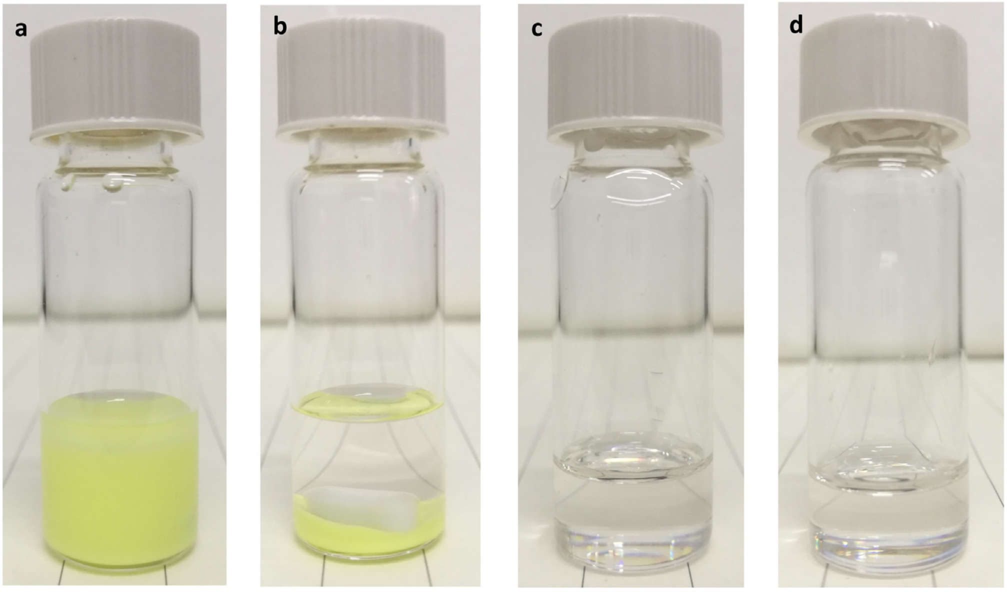

Supplementary Figure 19| The procedures of perovskite precursor preparation. **a,** The precursor solutions were made and stirred at 45°C overnight. **b,** The solution of (a) was stand for 4 hours at room temperature, precipitates were formed in the CsBr-rich solution. **c,** Top transparent solution was decanted and then **d,** solution of **c** was filtered for using.

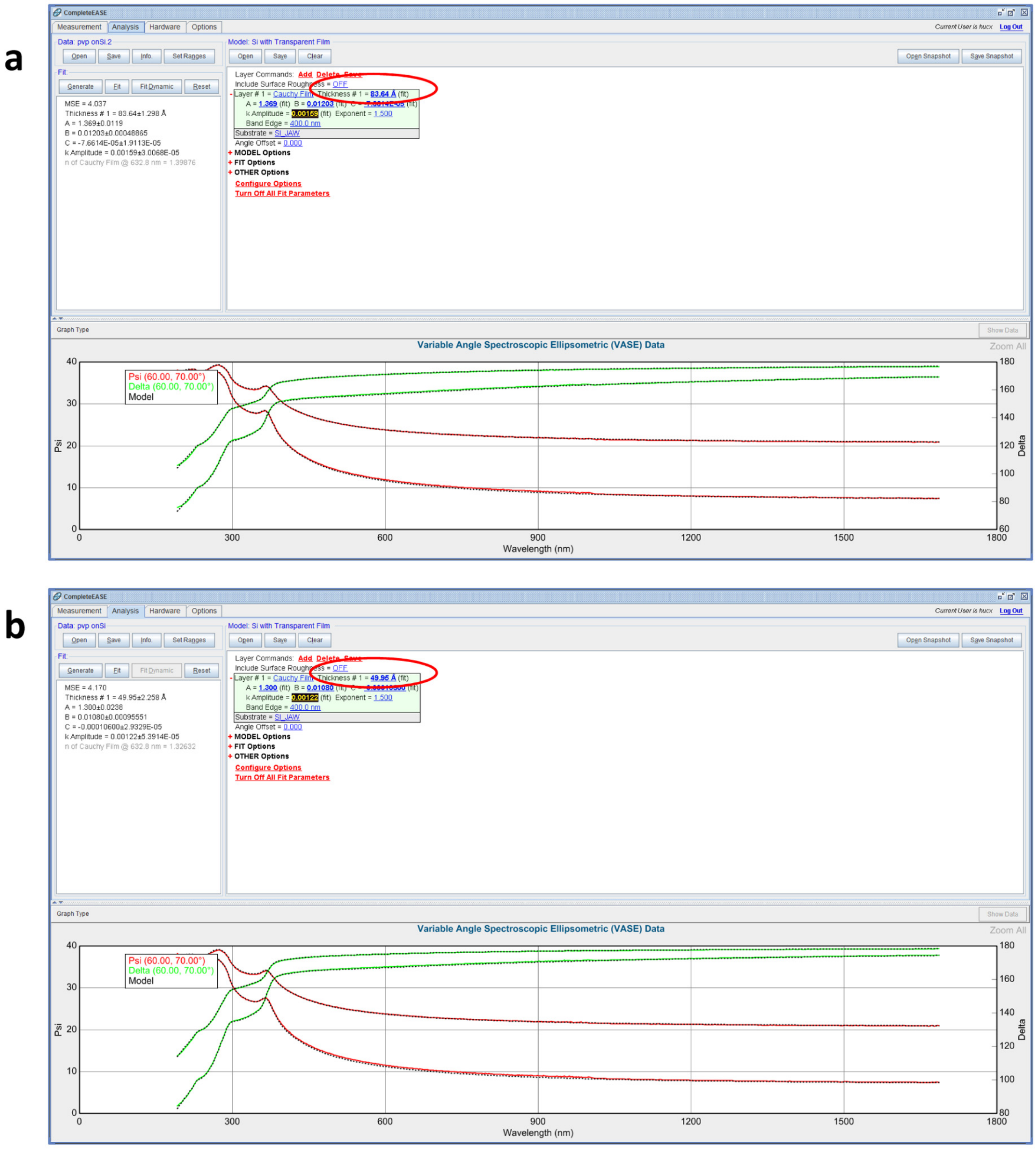

Supplementary Figure 20| The thicknesses of PVP layer **a**, before and **b**, after washing by DMSO solvent, which were measured by ellipsometer.

Supplementary Table 1| The band structure parameters of $CsPbBr_3$ and $Cs_{0.87}MA_{0.13}PbBr_3$.

Sample	E_g (eV)	W_F (eV)	VBM (eV)	E_V (eV)	E_C (eV)
$CsPbBr_3$	2.35	3.36	2.14	5.50	3.15
$Cs_{0.87}MA_{0.13}PbBr_3$	2.34	3.44	2.27	5.71	3.37

Supplementary Table 2| Summary of device performance with different thicknesses of the $CsPbBr_3$ layer.

$CsPbBr_3$ thickness	Voltage bias (V)	Current density (mA cm^{-2})	Luminance (cd m^{-2})	Current efficiency (cd A^{-1})	Maximum luminance (cd m^{-2})
100 nm	3.5	16.8	4460	26.5	64100
60 nm	4.5	132	13600	10.3	30800

Supplementary Table 3| Summary of device performance with different ratio of $CsBr:PbBr_2:MABr=x:1:0.1$ (x=2, 2.1, 2.2, 2.4).

$CsBr:PbBr_2:MABr$	Voltage bias (V)	Current density (mA cm^{-2})	Luminance (cd m^{-2})	Current efficiency (cd A^{-1})	Maximum Luminance (cd m^{-2})
2:1:0.1	4.0	34.7	7270	20.9	73700
2.1:1:0.1	4.0	55.0	10100	18.3	42100
2.2:1:0.1	4.0	18.2	6280	34.5	60400
2.4:1:0.1	4.5	59.8	5240	8.76	27300
